# Manipulation of ultrafast nonlinear optical response based on plasmon-induced magnetic anapole mode

**DOI:** 10.1039/d5ra06121k

**Published:** 2025-10-13

**Authors:** Yonglin He, Jingyu Wang, Weimin Yang, Shengjie jiang, Liqiang Zhuo

**Affiliations:** a School of Electronic Information, Zhangzhou Institute of Technology Zhangzhou 363000 China; b School of Physics and Information Engineering, Shanxi Normal University Taiyuan 030000 China wangjingyu@sxnu.edu.cn

## Abstract

Ultrafast all-optical switches are pivotal for advancing future optical communication and computing technologies. Plasmonic nanostructures, renowned for inducing strong Kerr nonlinear effects, have emerged as promising platforms for such devices. However, Kerr-type switches inherently face a trade-off between switching speed and modulation depth, posing a formidable challenge for their concurrent optimization. Herein, we propose a theoretically designed system comprising gold ellipsoid arrays, silica spacers, and gold films. This configuration achieves enhanced modulation depth by exploiting the strong optical confinement enabled by a magnetic anapole mode. Concurrently, the switching time is optimized through accelerated electron thermal equilibration *via* diffusion-mediated heat transport in hotspot regions. By systematically analyzing the spatiotemporal dynamics of electron temperature under varied pump wavelengths, we reveal the fundamental physical mechanisms underlying this performance enhancement. The proposed platform not only provides critical insights for ultrafast all-optical switching but also holds significant promise for advancing nanophotonic devices in optical information processing.

## Abstract 2

Ultrafast all-optical switches are pivotal for advancing future optical communication and computing technologies.

## Graphical abstract

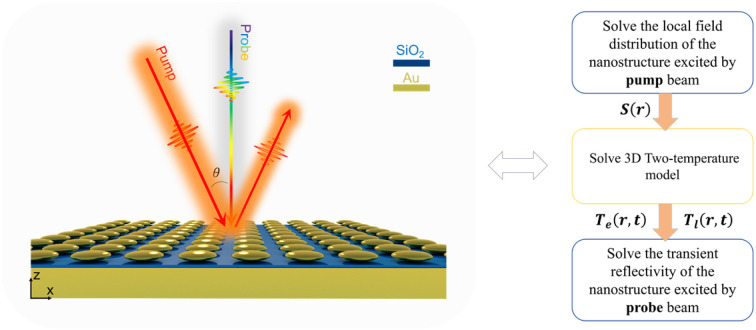

## Introduction

Optical switches are fundamental components for the manipulation of light fields, playing a pivotal role in systems that utilize light as an information carrier, such as optical computing and optical communications.^1–3^ The performance of an optical switch is primarily characterized by two crucial figures of merit: switching time and modulation depth. The former determines the operational bandwidth, while the latter dictates the signal-to-noise ratio. Based on the physical nature of their excitation source, optical switches can be categorized into several types, including thermo-optic,^4^ acousto-optic,^5^ magneto-optic,^6^ electro-optic,^7^ and all-optical switches.^8^ Among these, all-optical switches, which fundamentally rely on nonlinear optical effects to achieve light-field control, have garnered significant attention. Their operational principle involves using a control light pulse (the pump) to dynamically alter a material's optical properties, thereby modulating the transmission or reflection of a signal light pulse (the probe). This mechanism grants them a substantial advantage in terms of modulation speed over other switch types, with demonstrated potential for operation on femtosecond timescales.

Over the past decade, extensive research into all-optical switching has been conducted across a diverse range of material platforms. These include plasmonic noble metals^9–11^ (*e.g.*, gold and silver), two-dimensional (2D) materials,^12,13^ perovskites,^14^ and epsilon-near-zero (ENZ) materials.^15,16^ Among these platforms, plasmonic nanostructures have emerged as one of the most widely investigated systems for all-optical modulation. Their prominence is founded on a unique combination of advantages: the capacity to generate strong local field enhancements under plasmon resonance conditions,^17–20^ the extreme sensitivity of plasmon modes to the dielectric environment,^21,22^ and the intrinsically ultrafast dynamics of hot electrons.^23–26^ These characteristics provide a robust foundation for developing ultra-compact, high-performance all-optical switches.

Research efforts centered on plasmonic all-optical switching have largely focused on optimizing either the modulation depth or the switching time.^27^ To enhance modulation depth, the employed strategies include: geometric optimization of metallic nanostructures,^10,17^ integration of materials exhibiting enhanced optical nonlinearity or tunable phase-change characteristics,^28–31^ and design of engineered metamaterial architectures.^32,33^ To reduce switching times, the employed strategies include: optimizing plasmonic structures to accelerate thermal equilibration dynamics in hotspot regions,^11,17,23,24^ bypassing carrier relaxation limitations through two-photon absorption or second harmonic generation processes,^10,34^ and facilitating hot electron transfer to adjacent semiconductor layers.^35,36^ However, a majority of studies reveal a persistent trade-off: in a single system, enhancing the modulation depth often comes at the expense of the switching time, making their simultaneous optimization a formidable challenge. A common thread throughout these optimization methods is the critical role of nanostructure design. Therefore, developing a system architecture capable of concurrently optimizing both performance parameters is of paramount significance for the advancement of all-optical switching technology.

Herein, we theoretically design and investigate a hybrid system composed of a gold ellipsoid array, a silica spacer layer, and a continuous gold film. This architecture is engineered to support a rich variety of plasmonic modes, including a magnetic anapole mode and a magnetic toroidal dipole mode. Through the study of the system's transient optical properties, we uncover a pathway to overcome the conventional performance trade-off. We demonstrate that the magnetic anapole mode, due to its powerful confinement of the probe light field, can effectively increase the light–matter interaction and thus enhance the modulation depth. Specifically, we demonstrate a significant reflectivity modulation of 71.1% at a moderate pump fluence of 51 μJ cm^−2^. Simultaneously, the highly localized electric fields induced by the pump pulse enhance the contribution of electronic diffusion to the thermal equilibration of the hotspot. This process, which can be quantitatively described by established theoretical frameworks like the two-temperature model incorporating energy transport *via* diffusion equations accelerates the cooling of the electron system and thereby optimizes the switching time. The proposed system introduces a novel and effective strategy for designing high-performance plasmonic all-optical switches where both high modulation depth and ultrafast switching speed can be achieved simultaneously.

### Models and principle

**Fig. 1:**
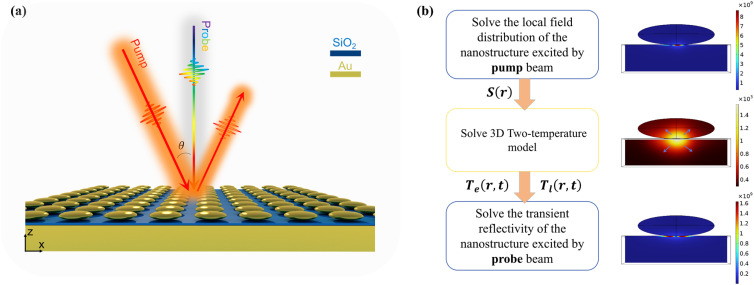
(a) Schematic of the Au ellipsoid array-SiO_2_–Au film system. Incident light propagates in the *xz*-plane, with the incidence angle measured relative to the *z*-axis. (b) The multiphysics simulation framework integrating electromagnetics and heat transfer.

The proposed system consists of an Au ellipsoid array, a silica spacer, and an Au film, as shown in Fig. 1a. Each Au ellipsoid has radii of 150 nm along the *x*- and *y*-axes and 40 nm along the *z*-axis, with a period of 400 nm. The silica spacer thickness is 4 nm.

Under illumination, localized and size-dependent electron modes are induced on the nanoparticle surfaces in the Au ellipsoid array-SiO_2_–Au film system. The mirror effect causes these electron modes to form anti-phase distributed mirror images in the Au film. The antisymmetric field distribution between each nanoparticle and its mirror image constitutes closed current loops, thereby generating a dominant magnetic dipole moment. Through efficient coupling of this magnetic dipole with the magnetic toroidal dipole mode, the excitation of the magnetic anapole state is ultimately achieved.

Both steady-state and transient optical simulations in this study were conducted using COMSOL Multiphysics software. For steady-state optical characterization, the electromagnetic wave frequency domain (EWFD) interface of the wave optics module was employed. The refractive indices of silica and air were set to 1.456 and 1, respectively. The optical parameters of gold were adopted from literature ref. 37. Periodic boundary conditions were applied in the *x* and *y* directions of the computational domain, while perfectly matched layers (PML) were implemented in the *z* direction. The incident light propagated from the *xz*-plane with an incident angle *θ* defined relative to the *z*-axis. Transient optical analysis utilized a multi-physics framework combining electromagnetics and heat transfer, as illustrated in Fig. 1b. The blue block represents the electromagnetic wave EWFD interface, while the yellow block denotes the solid heat transfer interface in the heat transfer module. The functionalities of each interface are specified within their respective blocks. The pump and probe beams were both linearly polarized, with their electric field vectors confined to the *xz*-plane of simulation. The pump pulse was set to a width of 80 fs (FWHM), and the probe pulse was configured to 80 fs, consistent with typical ultrafast optical experiments to facilitate subsequent experimental verification. The simulation process involved three sequential steps: first, solving the electromagnetic field distribution under pump excitation; second, converting the electromagnetic field into a heat source term for input into a 3D two-temperature model to resolve the spatial distribution and temporal evolution of electron temperature and phonon temperature. The 3D two-temperature model is expressed as:1
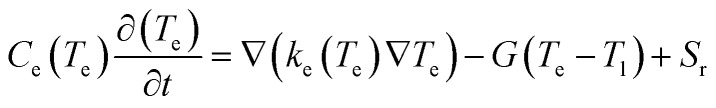
2
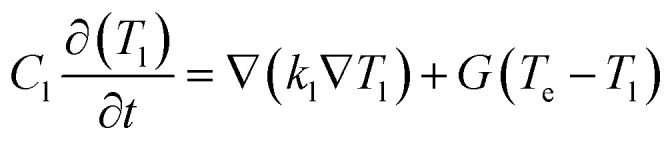
where *T*_e_, *C*_e_, and *k*_e_ denote the electron temperature, heat capacity, and thermal conductivity, respectively; *T*_l_, *C*_l_, and *k*_l_ denote the phonon temperature, heat capacity, and thermal conductivity, respectively; *G* denotes the electron–phonon coupling coefficient; and *S*_r_ denotes the heat source derived from electromagnetic simulations.^25^ To accurately resolve the transient thermal and optical dynamics, the numerical solver employed a gradient-based time stepping scheme, with a minimum step size of 1 fs to ensure convergence and capture the rapid evolution of electron temperature. The complex dielectric function of gold was modified based on a revised Drude model, where both the plasma frequency and the collision frequency were expressed as functions of *T*_e_ and *T*_l_, to simultaneously account for the effects of electron heating and lattice heating. Finally, the modified complex dielectric function was iteratively fed back to the electromagnetic solver for calculating time-resolved reflection spectra *R*.

The transient reflectivity change used in this study requires further conversion using the formula:

3 Δ*R*/*R* = (*R*_on_ − *R*_off_)/*R*_off_

where *R*_off_ is the reflectance of the unperturbed sample (before pump excitation) and *R*_on_ is the reflectance measured at delay time “t” after pump illumination. The right column of Fig. 1b shows one of the typical results obtained from these three steps.

## Results and discussion

**Fig. 2:**
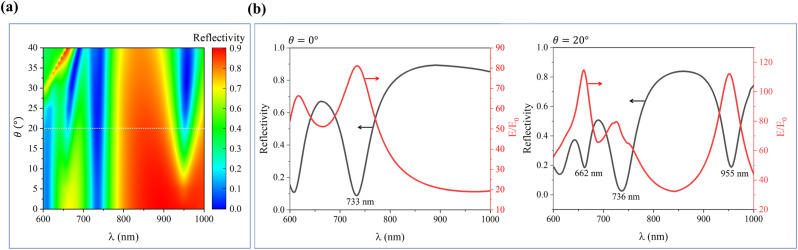
(a) Reflectance contour plot as a function of wavelength and incidence angle for the Au ellipsoid array-SiO_2_–Au film system. (b) Reflectance (black solid line) and electric field enhancement factor |*E*|/|*E*_0_| (red solid line) for the Au ellipsoid array-SiO_2_–Au film system at 0° (left panel) and 20° (right panel) incidence angles.

The steady-state optical properties of the Au ellipsoid array-SiO_2_–Au film system were first investigated under varying angles of incidence. Fig. 2a presents contour plots of reflectivity as a function of wavelength (*λ* = 600–1000 nm) and incident angle (*θ* = 0°–40°) for this system. The white dashed line indicates an incident angle of 20°. At this angle, four distinct spectral bands exhibit notably low reflectivity, suggesting the potential existence of four resonance modes located in the wavelength ranges of approximately 600–625 nm, 650–675 nm, 725–750 nm, and 925–975 nm, respectively. Due to limited information, the mode in the 600–625 nm range is not primarily discussed here. Fig. 2b displays the measured reflectivity (solid black line) and the electric field enhancement intensity |*E*|/|*E*_0_| (solid red line), where |*E*_0_| is the incident field strength, at *θ* = 0° and *θ* = 20°. Analysis of both the reflectivity minima and the associated electric field enhancement peaks reveals that at *θ* = 0°, a single dominant plasmonic mode exists, centered at 733 nm. At *θ* = 20°, three distinct plasmonic modes are observed, centered at 662 nm, 736 nm, and 955 nm.

**Fig. 3:**
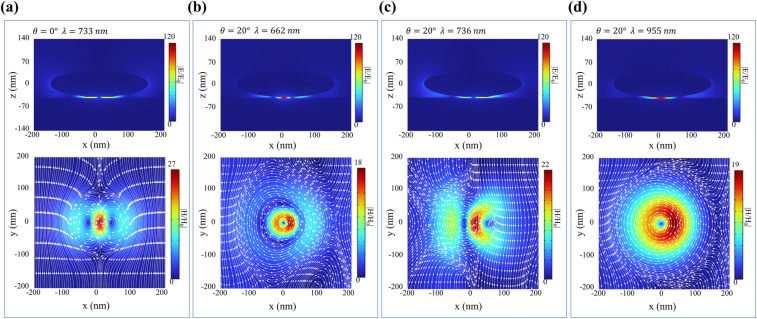
Electric field distribution in the *xz*-plane through the center of the Au ellipsoid (upper panels) and magnetic field distribution in the *xy*-plane on the upper surface of the SiO_2_ spacer (lower panels). (a) 0° incidence angle at 733 nm; (b)–(d) 20° incidence angle at 662 nm, 736 nm, and 955 nm, respectively.

To further elucidate the intrinsic mechanisms of these modes, Fig. 3 presents the electric field distribution in the *xz*-plane passing through the center of an Au ellipsoid (upper panels) and the magnetic field distribution in the *xy-*plane on the upper surface of the SiO_2_ spacer (lower panels). Fig. 3a illustrates the electromagnetic field characteristics at an incidence angle of 0° and a wavelength of 733 nm. At the central cross-section of the *xy*-plane, the magnetic field lines form two counter-rotating circles, generating two out-of-phase electric field points. This configuration leads to energy confinement within the SiO_2_ spacer, which is entirely consistent with the magnetic anapole mode reported in previous work.^38,39^Fig. 3b and d depict the electromagnetic field characteristics at an incidence angle of 20° and wavelengths of 662 nm and 955 nm, respectively. Both configurations exhibit magnetic field lines distributed along annular closed paths in the *xy*-plane, forming localized vortices with strong electric field concentrations at the vortex centers; these features correspond to magnetic toroidal dipole modes.^40,41^Fig. 3c shows the electromagnetic field characteristics at an incidence angle of 20° and a wavelength of 736 nm. Similar to Fig. 3a, at the central cross-section of the *xy*-plane, the magnetic field lines form two counter-rotating circles, and the electric field distribution also exhibits partial similarity to the case in Fig. 3a. However, this mode is not classified as a magnetic anapole mode because oblique incidence disrupts the optimal coherent coupling between the magnetic dipole and magnetic toroidal dipole modes.

**Fig. 4:**
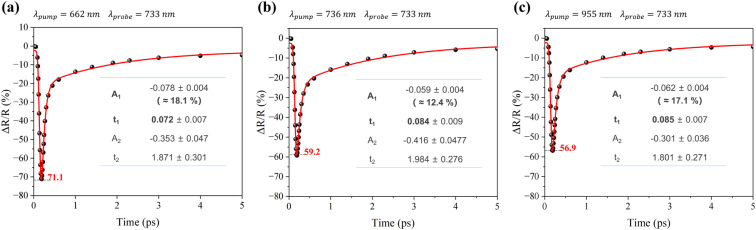
Transient reflectivity change (Δ*R*/R) *versus* time (*t*) under 733 nm probe light, with pump wavelengths of (a) 662 nm, (b) 736 nm, and (c) 955 nm, respectively.

To investigate the all-optical modulation capability of the Au ellipsoid array-SiO_2_–Au film system, transient reflectance characterization was performed (Fig. 4a–c), with the corresponding pump and probe wavelengths labeled in the top-left corner of each panel. The pump light was incident at 20° with wavelengths of 662 nm, 736 nm, and 955 nm, corresponding to the resonance wavelengths of the three plasmonic modes at this angle. The probe light was incident 0° with a wavelength of 733 nm, matching the resonance wavelength of the magnetic anapole mode at this angle. This non-coaxial pump-probe configuration offers two advantages: first, the pump light can resonantly excite a broader range of plasmonic modes, providing more opportunities to optimize the performance parameters of the all-optical switch; second, the probe light resonantly excites the magnetic anapole mode, maximizing its confinement of the probe light. In Fig. 4a–c, the black dots represent transient reflectance data, and the gray dashed lines indicate the minima of the transient reflectance Δ*R*/*R*_min_. The modulation depth (MD) is calculated using the standard formula: MD = |Δ*R*/*R*_min_|. Under excitation by pump light at wavelengths of 662 nm, 736 nm, and 955 nm, the system achieved modulation depths of 71.1%, 59.2%, and 56.9%, respectively. The energy fluence density of the pump light at all three wavelengths was set to 51 μJ cm^−2^ in this study, indicating that under the same pump fluence, the 662 nm pump excitation achieved the largest modulation depth. Compared to previously reported studies, this system exhibits advantages in modulation depth.^17,42^

To analyze the dynamic characteristics of the transient reflectance data in Fig. 4, the data were fitted with a sum of convoluted exponentials function:4
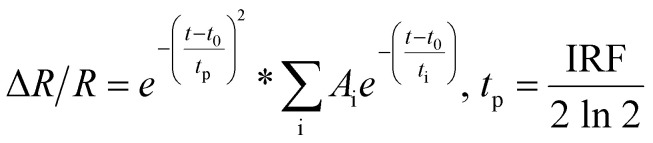
Where IRF is the width of instrument response function (full width half maximum), *t*_0_ is the time zero, *A*_i_ and *t*_i_ denote the amplitudes and decay times respectively, and * indicates the convolution operation. The red solid lines in Fig. 4 represent the fitting curves, and the fitting parameters are listed in the table. Here, *t*_1_ denotes the lifetime of the femtosecond transient component, and *A*_1_ represents its amplitude (normalized values in parentheses). Similarly, *t*_2_ and *A*_2_ correspond to the lifetime and amplitude of the picosecond transient component. For the same system, a shorter *t*_1_ and larger *A*_1_indicate a faster switching time and better optimization. Comparative analysis reveals that *t*_1_ is smallest and *A*_1_ is largest in Fig. 4a, meaning the 662 nm pump excitation achieves the optimal switching time. In summary, pump excitation at 662 nm enables simultaneous optimization of both modulation depth and switching time in this system.

For the Au ellipsoid array-SiO_2_–Au film system, its transient optical response arises from temperature-induced changes in the dielectric function, primarily driven by changes in electron temperature. The inherent localized electromagnetic field distribution of plasmonic nanostructures creates non-uniform spatial heating of electrons, leading to spatially non-uniform modifications of the dielectric function. Consequently, to elucidate the physical mechanisms underlying the observed differences in the magnetic anapole mode's transient optical response under three pump wavelengths, further analysis of the spatiotemporal electron temperature distribution is required.

**Fig. 5:**
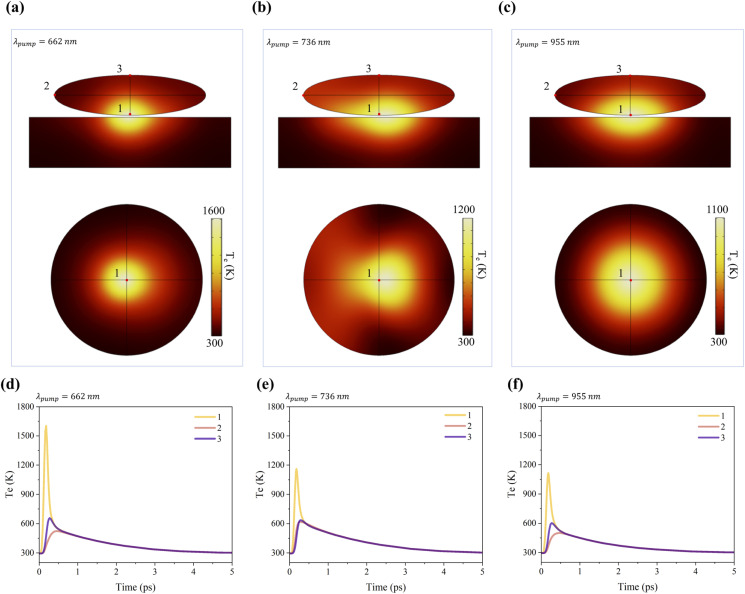
(a)–(c) Depict electron temperature distributions at *t* = 0.19 ps in the *xz*-plane through the center of the Au ellipsoid (upper panels) and on the lower surface of the Au ellipsoid (lower panels) under pump excitation at wavelengths of 662 nm, 736 nm, and 955 nm, respectively. (d)–(f) Show the temporal evolution of electron temperature at the three corresponding positions in (a)–(c).

Fig. 5a–c display the electron temperature distributions at *t* = 0.19 ps in the *xz-*plane passing through the center of an Au ellipsoid (upper panels) and on the lower surface of the Au ellipsoid (lower panels) under pump excitation at wavelengths of 662 nm, 736 nm, and 955 nm, respectively. Temperatures are indicated by the corresponding color bars. Since phonon temperature changes are significantly smaller than those of electrons and occur over much longer timescales—factors that have a minimal impact on the ultrafast transient optical response—phonon dynamics are not discussed in this study. The figures reveal pronounced spatial non-uniformity in the electron temperature distributions under all three pump conditions, with localized regions on the ellipsoid's lower surface exhibiting significantly higher temperatures than other areas. The high-temperature regions generated by 662 nm and 955 nm pump excitation demonstrate good symmetry; however, the former (662 nm) exhibits a smaller size and higher temperature, exceeding 1600 K. In contrast, the 736 nm pump excitation generates a high-temperature region with lower symmetry than the other two, with intermediate size and peak temperature. Higher temperatures correspond to a greater influence on the dielectric function, resulting in larger modulation depth. Thus, from the perspective of peak temperature, the differences in modulation depth across the three pump wavelengths can be explained, consistent with the principle that increased pump fluence enhances modulation depth.

Additionally, transient electron temperature data at three positions (bottom, edge, top) on the ellipsoid in Fig. 5a–c are shown in Fig. 5d–f, respectively. Fig. 5d and f exhibit similar temperature dynamics: position 1 has the highest peak temperature, followed by position 3, with position 2 being the lowest. In contrast, the temperature dynamics in Fig. 5e show closely matched peak temperatures at positions 2 and 3. This discrepancy arises from two factors: (1) The ellipsoid's larger radii along the *x-* and *y*-axes (compared to the *z*-axis) lead to greater diffusion-driven temperature rises at position 3 than at position 2. (2) The heat source is spatially concentrated at the ellipsoid's bottom in Fig. 5a and c but more broadly distributed in Fig. 5b, counteracting diffusion-induced differences. Comparing position 1 in Fig. 5d–f, Fig. 5d exhibits the highest electron temperature and the fastest relaxation rate, explaining why *t*_1_ is smallest and *A*_1_ is largest in Fig. 4a. This ultrafast component corresponds to the process where hot electrons rapidly transfer energy from hotspot regions to non-hotspot regions *via* diffusion, which constitutes the physical basis for achieving ultrafast switching speed.

Although our system has not yet attained hundred-femtosecond-scale switching times, we propose two complementary approaches to achieve this goal. The first approach involves structural size optimization; by increasing the dimensions of the metallic nanostructures, electron diffusion-mediated energy dissipation from hotspots is enhanced, enabling hundred-femtosecond-scale temperature reduction. The second method leverages the system's intrinsic dynamical properties, utilizing counteracting optical signals from the same pump pulse to cancel the electron–phonon scattering signal, thereby highlighting the ultrafast switching characteristics in the dynamic response.^11,43^ These two strategies address the challenge of overcoming rate limitations imposed by electron–phonon scattering through distinct mechanisms: one enhances ultrafast processes while the other suppresses slower processes, collectively providing effective solutions for achieving femtosecond-scale switching.

The proposed approaches are experimentally feasible. Although the fabrication of the Au ellipsoid array is challenging, it can be achieved through advanced nanofabrication processes, such as specially optimized electron-beam lithography or template-assisted techniques.^44^ The transient reflectance measurements can be implemented with a standard pump-probe spectroscopy setup. These factors confirm the practical viability of our proposed strategies. Compared to previously reported similar plasmonic structures, this study demonstrates significant advantages in several aspects: first, in terms of modulation depth, we achieved a modulation depth of up to 71.1%, which represents a substantial improvement over the values reported in literature for analogous nanostructures.^9,11,23^ Second, this study first observed position-dependent electron temperature distribution differences in ellipsoid structures, and revealed the coupling effect between diffusion mechanisms and the spatial distribution of heat sources. This discovery provides new perspectives for understanding energy transfer mechanisms in plasmonic systems. Furthermore, our proposed dual-strategy approach (structural optimization and signal cancellation) offers a more comprehensive solution for achieving ultrafast switching compared to traditional single-method temperature regulation approaches.

## Conclusions

In conclusion, we have developed an all-optical modulation platform comprising gold ellipsoid arrays, SiO_2_ spacers, and gold films. Transient reflectance measurements under three distinct pump wavelengths revealed that 662 nm excitation enables the system to attain both maximum modulation depth and fastest switching time. Furthermore, by investigating the spatiotemporal evolution and relaxation dynamics of the transient electron temperature distribution, we revealed the fundamental physical mechanisms governing the observed variations in optical response under different pump conditions. This work presents a novel method to overcome the inherent trade-off between modulation depth and switching speed in plasmonic all-optical switches, thereby establishing a versatile design paradigm for the development and optimization of next-generation high-performance photonic devices.

## Conflicts of interest

There are no conflicts to declare.

## Data Availability

All data needed to evaluate the conclusions in the paper are present in the paper.
